# Pilot trial on the efficacy and safety of pantethine in children with pantothenate kinase-associated neurodegeneration: a single-arm, open-label study

**DOI:** 10.1186/s13023-020-01530-5

**Published:** 2020-09-14

**Authors:** Xuting Chang, Jie Zhang, Yuwu Jiang, Bufan Yao, Jingmin Wang, Ye Wu

**Affiliations:** 1 Department of Pediatrics, Peking University First Hospital, No.1, Xi’an Men Street, West District, Beijing, 100034 China; 2 Department of Clinical Pharmacy, School of Pharmaceutical Sciences, Shandong University, Jinan, China

**Keywords:** Pantethine, Pantothenate kinase-associated neurodIegeneration, Efficacy, Safet**y**

## Abstract

**Objective:**

This study aimed to explore the efficacy and safety of pantethine in children with pantothenate kinase-associated neurodegeneration (PKAN).

**Methods:**

A single-arm, open-label study was conducted. All subjects received pantethine during the 24-week period of treatment. The primary endpoints were change of the Unified Parkinson’s Disease Rating Scale (UPDRS) I–III and Fahn–Marsden (FM) score from baseline to week 24 after treatment.

**Results:**

Fifteen children with PKAN were enrolled, and all patients completed the study. After 24 weeks of treatment with pantethine at 60 mg/kg per day, there was no difference in either UPDRS I–III (t = 0.516, *P* = 0.614) or FM score (t = 0.353, *P* = 0.729) between the baseline and W24. Whereas the rates of increase in UPDRS I-III (Z = 2.614, *p* = 0.009) and FM scores (Z = 2.643, *p* = 0.008) were slowed. Four patients (26.7%) were evaluated as “slightly improved” by doctors through blinded video assessment. Patients with lower baseline UPDRS I–III or FM scores were more likely to be improved. The quality of life of family members improved after pantethine treatment, evaluated by PedsQL TM 2.0 FIM scores, whereas the quality of life of the patients was unchanged at W24, evaluated by PedsQL TM 4.0 and PedsQL TM 3.0 NMM. Serum level of CoA was comparable between baseline and W24. There was no drug related adverse event during the study.

**Conclusions:**

Pantethine could not significantly improve motor function in children with PKAN after 24 weeks treatment, but it may delay the progression of motor dysfunction in our study. Pantethine was well-tolerated at 60 mg/kg per day.

**Trial registration:**

Clinical trial registration number at www.chictr.org.cn:ChiCTR1900021076, Registered 27 January2019, the first participant was enrolled 30 September 2018, and other 14 participants were enrolled after the trial was registered.

## Introduction

Pantothenate kinase-associated neurodegeneration (PKAN, OMIM#234200, formerly known as Hallervorden–Spatz syndrome), an autosomal recessive disease caused by mutations in the *PANK2* [1], is classified into two subtypes, namely, classic and atypical phenotype [2]. Approximately two-thirds of patients are classic phenotype, which is usually characterized by dystonia before 10 years of age and loss of ambulation 10–15 years after the disease onse t[3]. The mutations in *PANK2* result in decreased activity of pantothenic acid kinase 2, which is essential for the biosynthesis of coenzyme A (CoA) using pantothenic acid, thereby leading to the reduction of CoA and iron accumulation in specific brain regions [4]. The estimated prevalence is 1–2/1,000,000 [5]. Therapies tried in patients with PKAN included deep brain stimulation (DBS), deferiprone and fosmetpantotenate (RE-024). Currently there is no promising disease-modifying therapy for PKAN.

Pantethine is a marketed drug used to treat hyperlipidemia. Studies on a *PANK2* knockout *Drosophila* model [6] and a *PANK2* knockout mouse model [7] showed that pantethine could aid mitochondrial dysfunction, increase CoA levels in tissues, and rescue the neuromuscular phenotypes. In this study, we aimed to explore the efficacy and safety of the re-purposed drug treatment of pantethine in children with PKAN.

## Methods

### Study design

A single-arm, open-label study was conducted. All subjects received pantethine during the 24-week period of treatment. This study was approved by the Ethics Committee of Peking University First Hospital and registered at the Chinese Clinical Trial Registry (ChiCTR1900021076). All the parents or legal guardians of children signed the written informed consent forms before any procedure was done.

### Participants

Patients who met all the following criteria were enrolled: (1) aged 3–16 years who showed progressive dystonia; (2) “eye-of-the-tiger” sign in brain magnetic resonance imaging; (3) pathogenic or likely pathogenic variants identified in *PANK2*. Patients were excluded if they met any of the following criteria: (1) patients who participated in other clinical trials; (2) with severe liver damage (alanine aminotransferase or aspartate aminotransferase > 3 times the upper limit of normal value);(3) severe renal damage with glomerular filtration rate < 60 ml/min;(4) with cardiovascular system diseases, including cardiac dysfunction or arrhythmia; (5) abnormalities in blood system (hemoglobin < 60 g/L, platelet < 50 × 10^9^/L, neutrophil < 1.0 × 10^9^/L); and (6) patients who underwent DBS in the past 6 months.

### Procedure

The study lasted for 24 weeks. Patients received pantethine through oral administration (capsules, 450 mg per serving, Jarrow Formulas, USA). The dose started at 20 mg/kg per day, gradually increased to 60 mg/kg per day within 4 weeks and maintained at 60 mg/kg per day (divided into two dosage) until the end of the study. During the study, the dosage of other drugs taken by patients at baseline remained unchanged. Patients were contacted weekly by phone to find out if there were any drug related adverse events in the first 5 weeks and were visited at week 12 (W12) and 24 (W24).

**Fig. 1:**
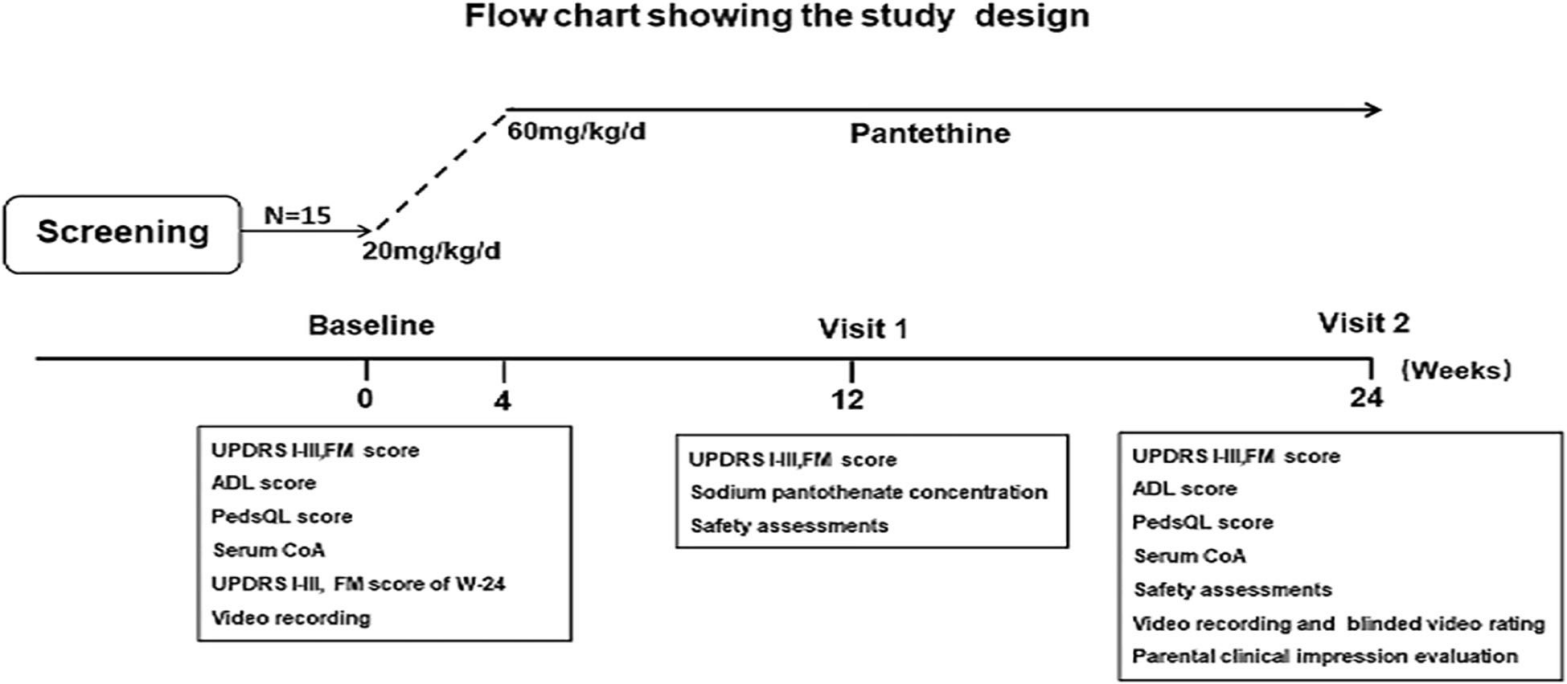
Flow chart of the study design. The treatment period lasted for 24 weeks. The dose of pantethine starts at 20 mg/kg per day, gradually increases to 60 mg/kg per day within 4 weeks and maintain at 60 mg/kg per day until the end of the study. The UPDRS I–III and FM at W-24 (24 weeks before treatment) and W0 (baseline) were scaled at baseline. At baseline(W0), ADL and PedsQL were scaled, motion videos were recorded, and serum CoA levels were measured. At W12, the UPDRS I–III and FM scores were re-scaled, sodium pantothenate concentrations were measured. At W24, the UPDRS I–III and FM scores were re-scaled, ADL, PedsQL, parental clinical impression evaluation and blinded video rating by doctors were scaled. Serum CoA levels were also measured at W24. Safety assessments were conducted at each visit through data

The Unified Parkinson’s Disease Rating Scale I-III (UPDRS I-III) and Fahn–Marsden Scale (FM) at W-24 (24 weeks before treatment) were retrospectively assessed at baseline. At baseline(W0), UPDRS I-III and FM scores, Activities of daily living (ADL) and Pediatric Quality of Life Inventory (PedsQL) were scaled, motion videos were recorded, and serum CoA levels were measured. At W12 and W24, the UPDRS I-III and FM scores were re-scaled. ADL, PedsQL, parental clinical impression evaluation and blinded video rating were scaled at W24 (Fig. 1).

### Assessments of the efficacy and safety

Assessments of efficacy were conducted using the following measures: (1) UPDRS I-III was used to assess motor symptoms, including mental behavior, daily activity, and motor sections by generating individual scores and a total score (0–124); (2) FM scale was used to measure the severity of dystonia in nine body regions (eyes, mouth, neck, trunk, swallowing, speech, and each upper and lower extremities) by generating individual scores and a total score (0–120); (3) ADL scale was used to measure the patients’ ability of daily living, the higher the score, the better the ability of self-care; (4) PedsQL scale was used to measure quality of life, which includes PedsQL4.0 (Generic Core Scales), PedsQL3.0 (Neuromuscular Module), and PedsQL2.0 (Family Impact Module). The first two were used to assess patients’ quality of life. PedsQL2.0 was used to evaluate the quality of life of the families; (5) Parental clinical impression scale, which is a subjective instrument consists of a single question, was used to ask parents to rate their children’s condition on a scale from 1 (markedly better) to 5 (markedly worse) compared with how they felt at the baseline; (6) Blinded video rating was independently performed by two pediatric neurologists. Conflicts in opinions were resolved by consulting another neurologist to make a decision. Patients’ condition at W24 was rated on a scale from 1 (markedly better) to 5 (markedly worse) compared with how they felt at baseline through watching the videos (the actions included opening and closing eyes, opening and closing mouth, turning head, eye movements, tapping the index finger with the thumb, stretching and bending arms, placing both arms flexed at the elbow in front of the chest, stamping the foot, finger–nose test, turning body, walking, eating something, writing, and speaking).

Safety assessments were conducted at each visit through data collection of adverse events and clinical laboratory tests including liver function, renal function, and complete blood count.

The primary endpoints were the change of UPDRS I-III and FM scores from baseline to W24 after pantethine treatment. The secondary endpoints were changes in ADL score, PedsQL score, parental clinical impression scale, as well as blinded video rating from baseline to week 24. The increase rates of UPDRS I-III and FM scores 24 week after and before pantethine treatment were also compared.

### Measurement of serum CoA

Blood was extracted at baseline and W24. The serum was immediately centrifuged (4000 rpm/min, 10 min) and stored at − 20 °C until CoA was measured. Serum CoA was measured using a colorimetric-based kit (Abcam, ab102504) in accordance with the manufacturer’s instructions. A CoA standard curve was generated on the basis of optical density and serial dilution of CoA standard. CoA levels in test samples were calculated using a calibration curve.

### Measurement of sodium pantothenate

Blood was extracted two and five hours after taking pantethine at W12. Sodium pantothenate concentrations were determined by high-performance liquid chromatography coupled with mass spectrometry approach (LC-MS/MS), with ceftiofur as an internal standard. Negative electrospray ionization tandem mass spectrometry with multiple reaction monitoring mode was choose for the detection. Protein precipitation was applied with acetonitrile in the extraction process. The calibration curve was linear over the range of 0.05 to 50 μg/mL. The lower limit of quantification (LLOQ) was 0.05 μg/mL. The accuracy of low, middle and high concentration controls was 101.9, 114.1 and 114.2%, respectively.

### Statistical analysis

Descriptive statistics were used to summarize the collected data. Categorical variables were summarized on the basis of frequencies and percentages. Continuous variables were summarized on the basis of mean, median, standard deviations, and minimum and maximum values. The change of UPDRS I-III, FM, ADL, and PedsQL scores from baseline to week 24 were compared through paired t test. The increase rate of UPDRS I-III and FM scores before and after treatment were compared using the Wilcoxon rank sum test. The correlation between blinded video rating and initial UPDRS I-III/FM scores at baseline was tested through one-way ANOVA. Spearman’s correlation was used to compare the correlation between sodium pantothenate concentrations and change of UPDRS I–III/FM scores. The correlation between parental clinical impression scale/ blinded video rating by doctors and the change of UPDRS I–III/FM scores during treatment of pantethine was tested through Kendall’s tau-b correlation coefficient. All statistical analyses were conducted on SPSS (version 20.0). *P* < 0.05 was considered statistically significant.

## Results

### Clinical characteristics of patients

Fifteen children composed of seven males and eight females were enrolled, who were referred from eight hospitals in mainland China, and no patient withdrawal during the 24-week study. The median age at onset was 3.0(1.0–8.0) years. The median age and disease duration at baseline were 10.0 (3.5–13.6) and 5.3 (0.8–12.1) years, respectively. All patients enrolled were classic PKAN. For the initial symptom, ten patients (10/15, 66.7%) showed dystonia in lower limbs, three patients (3/15, 20.0%) showed dystonia in upper limbs, two (2/15, 13.3%) presented with developmental delay. At the baseline, five patients (5/15, 33.3%) lost the ability of independent ambulance. All patients showed “eye-of-tiger sign” in the brain MRI. The baseline medications taken by the patients included trihexyphenidyl hydrochloride, baclofen, levodopa, clonazepam, clozapine and mantadine (Table 1).

**Table 1:** Clinical and genetic characteristics of the patients

Patient ID	Sex	Age at enrollment (years)	Age at onset (years)	Symptoms at W-24	Symptoms at W0	Baseline medication	Mutation in *PANK2*	
							Change of nucleotide	Change of amino acid
01	M	10.0	8.0	DUL,DLL	DUL,DLL	Levodopa, Baclofen	c.970G>T/c.970G>T	p.D324Y/ p.D324Y
02	F	12.6	1.5	DUL,DLL, BS	DUL,DLL, BS	/	c.790C>T/−	p.R264W/ -
03	M	11.8	1.5	DUL,DLL, BS	DUL,DLL, BS, LOA	Trihexyphenidyl hydrochloride	c.1355A>G/c.1555 T>C	p.D452G/ p.F519L
04	F	7.9	7.1	DUL,DLL	DUL,DLL	Levodopa, Baclofen	c.856 C>T/c. 1502 T>A	p.R286C/p.I501N
05	F	8.9	4.0	DUL,DLL, BS	DUL,DLL, BS	/	c.1324G>T/c.1324G>T	p.D442Y/p.D442Y
06	M	9.0	2.0	DUL,DLL, BS, LOA	DUL,DLL, BS, LOA	Baclofen, Clonazepam, Amantadine, Clozapine Trihexyphenidyl hydrochloride	c.650A > G/c.1341 T > G	p.D217G/ p.D447E
07	M	10.2	7.8	DUL,DLL, BS	DUL,DLL, BS	Trihexyphenidyl hydrochloride, Baclofen	c.1502 T > A/c.833G > A	p.I501N/p.R278H
08	M	3.5	1.6	DUL,DLL	DUL,DLL	Trihexyphenidyl hydrochloride, Baclofen	c.1355A > G/c.1355A > G	p.D452G/ p.D452G
09	F	12.3	6.0	DUL,DLL	DUL,DLL	/	c.981 + 3A > G/ c.970G > T	Splice/p.D324Y
10	F	13.6	1.5	DUL,DLL, BS, LOA	DUL,DLL, BS, LOA	Baclofen, Tiapride, Levodopa, Trihexyphenidyl hydrochloride	c.595C>T/c.1351C>T	p.Q199X/p.R451X
11	F	10.3	5.0	DUL,DLL, BS	DUL,DLL, BS	Trihexyphenidyl hydrochloride, Clonazepam	c.1213 T>C/ c.510-522del	p.Y405H/p.A170Afs*31
12	F	8.3	2.0	DUL,DLL, BS	DUL,DLL, BS	Trihexyphenidyl hydrochloride, Baclofen, Levodopa	c.1355A>G/c.1355A>G	p.D452G/p.D452G
13	M	4.5	1.0	DUL,DLL, BS, LOA	DUL,DLL, BS, LOA	Levodopa, Clonazepam	c.1351C>T/c.1355A>G	p.R451X/p.D452G
14	M	5.6	2.0	DUL,DLL, BS	DUL,DLL, BS	/	c.644-645delGAinsAT/c.1151C>G	p.D215delIinsD/p.P384R
15	F	10.1	3.0	DUL,DLL	DUL,DLL, LOA	Trihexyphenidyl hydrochloride	c.1082A>G/ c.1355A>G	p.Y361C/p.D452G

*M*:male, *F* female, *LOA* loss of independent ambulance, *DLL* dystonia in lower limbs, *DUL* dystonia in upper limbs, *BS* bulbar symptoms,

“-”:not identified,“/”: no medication was taken at baseline, the transcript ID is NM_153638

### Primary endpoints of efficacy

To determine whether pantethine treatment could improve the motor function, we compared the UPDRS I-III scores (the assessment of mental behavior, daily activity, and motor function) and FM scores between the W24 and W0 in these patients. There was no significant difference between the baseline (W0) and W24 after the treatment, in either total UPDRS I-III scores or individual score of UPDRS I, II and III (Table 2). Moreover, the FM scores did not change significantly at W24 compared to W0 (Table 3). The results suggested that there was no significant improvement in motor handicap after 24 weeks of pantethine.

**Table 2:** Score of UPDRS I-III at W-24, baseline(W0) and W24

Patient	Score of UPDRS I					Score of UPDRS II					Score of UPDRS III					Total score of UPDRS I-III				
ID	W-24	W0	W24	∆_W 24-W0_	∆_W0-W-24_	W-24	W0	W24	∆_W 24-W0_	∆_W0-W-24_	W-24	W0	W24	∆_W24-w0_	∆_W0-W-24_	W-24	W0	W24	∆_W 24-w0_	∆_W 0-W-24_
1	0.0	8.0	1.0	−7.0	8.0	10.0	16.0	23.0	7.0	6.0	13.0	20.0	27.0	7.0	7.0	23.0	44.0	51.0	7.0	21.0
2	0.0	1.0	1.0	−0.0	1.0	21.0	26.0	25.0	−1.0	5.0	39.0	37.0	35.0	−2.0	−2.0	60.0	64.0	61.0	−3.0	3.0
3	0.0	1.0	0.0	−1.0	1.0	25.0	42.0	37.0	−5.0	17.0	42.0	57.0	50.0	−7.0	15.0	67.0	100.0	87.0	−13.0	33.0
4	0.0	0.0	0.0	0.0	0.0	4.0	15.0	11.0	−4.0	11.0	7.0	11.0	12.0	1.0	4.0	11.0	26.0	23.0	−3.0	15.0
5	0.0	0.0	0.0	0.0	0.0	19.0	19.0	22.0	3.0	0.0	21.0	29.0	33.0	4.0	8.0	40.0	48.0	55.0	7.0	8.0
6	0.0	1.0	1.0	0.0	1.0	35.0	38.0	37.0	−1.0	3.0	58.0	63.0	60.0	−3.0	5.0	93.0	102.0	98.0	− 1.0	9.0
7	1.0	1.0	0.0	−1.0	0.0	22.0	25.0	29.0	4.0	3.0	33.0	39.0	47.0	8.0	6.0	56.0	65.0	76.0	11.0	9.0
8	1.0	1.0	1.0	0.0	0.0	18.0	20.0	17.0	−3.0	2.0	22.0	19.0	13.0	−6.0	−3.0	41.0	40.0	31.0	−9.0	−1.0
9	1.0	2.0	2.0	0.0	1.0	7.0	13.0	10.0	−3.0	6.0	13.0	23.0	25.0	2.0	10.0	21.0	38.0	37.0	−1.0	17.0
10	0.0	0.0	1.0	1.0	0.0	28.0	28.0	28.0	0.0	0.0	49.0	52.0	48.0	−4.0	3.0	77.0	80.0	77.0	−3.0	3.0
11	1.0	2.0	0.0	−2.0	1.0	5.0	16.0	9.0	−7.0	11.0	10.0	19.0	13.0	−6.0	9.0	16.0	37.0	22.0	−15.0	21.0
12	1.0	1.0	1.0	0.0	0.0	25.0	33.0	33.0	0.0	8.0	41.0	51.0	57.0	6.0	10.0	67.0	85.0	91.0	6.0	18.0
13	0.0	0.0	0.0	0.0	0.0	27.0	32.0	33.0	1.0	5.0	36.0	43.0	47.0	4.0	7.0	63.0	75.0	80.0	5.0	12.0
14	0.0	0.0	0.0	0.0	0.0	25.0	27.0	35.0	8.0	2.0	35.0	37.0	55.0	18.0	2.0	60.0	64.0	90.0	26.0	4.0
15	2.0	2.0	0.0	−2.0	0.0	11.0	25.0	30.0	5.0	14.0	16.0	39.0	46.0	7.0	23.0	29.0	66.0	76.0	10.0	37.0
M± SD	/	/	/	/	/	18.8 ± 9.4	25.0 ± 8.7	25.3 ± 9.7	/	/	29.0 ± 15.6	35.9± 15.6	37.9 ± 16.6	/	/	48.3 ± 24.5	62.3 ± 23.3	63.7± 25.8	/	/
t/Z		1.087^a^		2.354^b^			0.234^a^		2.451^b^			1.096^a^		2.311^b^			0.516^a^		2.614^b^	
P		0.071^a^		0.019^b^			0.818^a^		0.014^b^			0.291^a^		0.021^b^			0.614^a^		0.009^b^	

“/” means no statistical analysis was performed, “a” means difference between W24 and W0, Paired t test and Wilcoxon rank sum test were performed

“b” means difference of the rate of increase before and after treatment, Wilcoxon rank sum test was performed

**Table 3:** FM score at baseline(W0) and W24

ID	W-24	W0	W24	∆_W24-W0_	∆_W0-W-24_
1	24.0	44.0	42.0	−2.0	20.0
2	63.0	72.0	61.0	−11.0	9.0
3	55.0	75.0	66.0	−9.0	20.0
4	8.0	12.0	9.0	−3.0	4.0
5	40.0	50.0	44.0	−6.0	10.0
6	90.0	102.0	94.0	−8.0	12.0
7	51.0	71.0	75.0	4.0	20.0
8	18.0	18.0	16.0	−2.0	0.0
9	15.0	31.0	35.0	4.0	16.0
10	65.0	60.0	61.0	1.0	−5.0
11	6.0	24.0	15.0	−9.0	18.0
12	60.0	70.0	76.5	6.5	10.0
13	46.0	49.0	56.0	7.0	3.0
14	36.0	51.0	74.0	23.0	15.0
15	23.0	49.0	67.0	18.0	26.0
**M ± SD**	40.0 ± 24.3	51.9 ± 24.3	52.8 ± 25.3	/	/
**t/Z**		0.353^a^		2.643^b^	
**P**		0.729^a^		0.008^b^	

“/” means no statistical analysis was performed

“a” means difference between W24 and W0, Paired t test was performed

“b” means difference of the rate of increase before and after treatment, Wilcoxon rank sum test was performed

### Secondary endpoints of efficacy

#### Rate of the increase in UPDRS I-III and FM scores 24 weeks before and after pantethine treatment

**Fig. 2:**
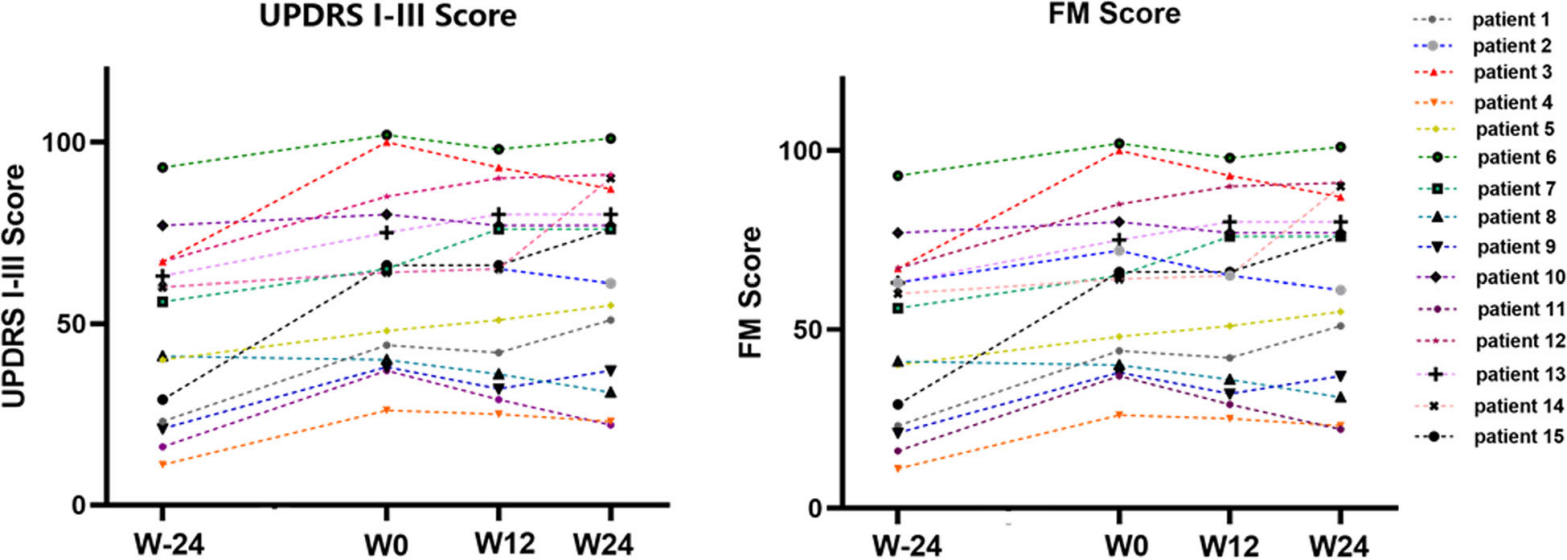
UPDRS I-III and FM score of 15 patients at W-24, W0, W12 and W 24. Each line in the figure represented an individual

To clarify whether pantethine could slow the progression of motor dysfunction in children with PKAN, we compared the rate of increase in score of UPDRS I-III and FM 24 week before and after the pantethine treatment. The rates of increase in the total score of UPDRS I-III, individual score of UPDRS I-III and FM scores were significantly slowed after the treatment (Table 2, Table 3, Fig. 2). The results indicated that pantethine may slow the progression of motor dysfunction.

#### Parental clinical impression scale and blinded video rating by doctors at W24

The subjective clinical impression was scaled by the parents or legal guardians of the fifteen children at W24. “Slightly improved” was rated by three parents (3/15, 20.0%), “No change” in three (3/15, 20.0%), “slightly deteriorated” in seven (7/15, 46.7%), and “markedly worse” in two parents (2/15, 13.3%) (Table 4). Parental clinical impression scale was consistent with the change of UPDRS I-III and FM scores during the 24-week treatment. (Kendall’s tau-b = 0.601, *P* = 0.005/ Kendall’s tau-b = 0.508, *P* = 0.017).

**Table 4:** The secondary endpoints at baseline and W24

Patient ID	ADL Score		PedsQL TM 4.0 Score		PedsQL TM 3.0 NMM Score		PedsQL TM 2.0FIM Score		Parental Clinical Impression Scale	Blinded video rating
	W0	W24	W0	W24	W0	W24	W0	W24		
1	75.0	65.0	61.8	23.0	60.0	62.0	50.0	58.8	Slightly worse	Slightly worse
2	10.0	40.0	30.0	27.8	39.0	36.0	71.5	50.0	Same	Slightly better
3	25.0	15.0	36.0	39.5	48.0	49.0	58.8	69.0	Same	Same
4	75.0	85.0	52.8	88.0	75.0	92.0	85.8	85.3	Slightly better	Slightly better
5	85.0	80.0	67.5	56.5	62.0	56.0	63.5	81.8	Slightly worse	Slightly worse
6	10.0	10.0	29.0	25.0	35.0	30.0	50.0	73.8	Slightly worse	Same
7	45.0	45.0	42.5	45.0	52.0	40.0	29.0	45.3	Slightly worse	Markedly worse
8	50.0	55.0	61.3	60.0	/	/	55.5	53.5	Slightly better	Slightly better
9	95.0	95.0	59.8	58.8	68.0	72.0	70.3	75.8	Slightly worse	Slightly worse
10	25.0	25.0	16.0	42.5	38.0	62.0	41.3	40.0	Same	Same
11	85.0	90.0	35.0	73.9	77.0	81.0	50.0	59.5	Slightly better	Slightly better
12	50.0	20.0	23.8	38.3	31.0	44.0	55.5	79.8	Markedly worse	Markedly worse
13	35.0	30.0	65.8	35.0	/	/	41.3	40.0	Slightly worse	Slightly worse
14	50.0	40.0	35.8	22.5	40.0	45.0	58.8	60.8	Slightly worse	Slightly worse
15	50.0	35.0	36.0	44.8	52.0	49.0	32.5	46.5	Markedly worse	Markedly worse
M ± SD	49.3 ± 28.1	53.0 ± 25.1	55.2 ± 18.1	45.3 ± 19.1	52.1 ± 15.4	55.2 ± 18.1	54.2 ± 15.1	61.3 ± 15.4	/	/
t	1.361^a^		0.332^a^		1.142^a^		2.306^a^		/	/
P	0.195^a^		0.745^a^		0.276^a^		0.037^a^		/	/

“/” means no statistical analysis was performed

“a” means difference between W24 and W0, Paired t test was performed

Pediatricians blindly compared the videos of motor action for each patient at W24 and W0. Four patients (4/15, 26.7%) were rated as “slightly improved”, three (3/15, 20.0%) “unchanged”, five (5/15, 33.3%)” slightly worse”, and three patients (3/15, 20.0%) were “markedly worse”. The blinded video rating by doctors of 12 patients (12/15, 80.0%) was consistent with the parental clinical impression scale (Table 4). Blinded video rating by doctors was consistent with the change of UPDRS I-III and FM scores and parental clinical impression scale (Kendall’s tau-b = 0.666, *P* = 0.002/ Kendall’s tau-b = 0.587, *P* = 0.005/ Kendall’s tau-b = 0.876, *P*<0.001). The UPDRS I-III and FM scores of all patients who were assessed as “slightly improved” in blinded video rating declined after pantethine treatment.

Subsequent analysis showed that blinded video rating was significantly associate with the UPDRS I-III (F = 8.708, *P* = 0.003) and FM scores (F = 4.401, *P* = 0.029) at the baseline. In patients with lower baseline UPDRS I-III or FM scores, they were more likely to be assessed as “slightly improved”, which may indicated that patients with relatively mild motor handicap at baseline were more likely to improve after the treatment.

#### ADL scale and PedsQL scale at baseline and W24

There was no difference between the W0 and W24 in ADL scale, which was used to measure the patients’ ability of daily living. With regard to the quality of life, there was an increase in PedsQL TM 2.0 FIM scores at W24, suggesting the improvement of the quality of life of the family members. Whereas, the quality of life of the patients remained unchanged, which was evaluated by PedsQL TM4.0 and PedsQL TM 3.0 NMM (Table 4).

### Serum CoA

The serum level of CoA at the baseline and W24 were 26.1 (9.3–122.4) nmol/mL and 14.3(16.5–162.0) nmol/mL, respectively. No significant difference was found before and after 24 weeks of treatment (Z = 1.014, *P* = 0.31).

### Sodium pantothenate in plasma

Sodium pantothenate in plasma was measured in 13 subjects. The plasma sodium pantothenate concentrations were 1.56 ± 0.91 μg/ml and 1.04 ± 0.34 μg/ml at 2 h and 5 h after taking pantethine. There was no significant correlation between the concentration of sodium pantothenate at two hours after taking pantethine and the efficacy, represented as the change of UPDRS I-III or FM score (rs = 0.094, *P* = 0.76/rs = 0.179, *P* = 0.558).

### Safety

Pantethine was well tolerated in all children during the 24-week treatment. Two patients showed mild nausea and poor appetite. Liver and renal function, as well as blood routine remained normal during the study period.

## Discussion

In this pilot study on 15 children with PKAN, we did not find 24 weeks of pantethine could significantly improve the motor function, but we found that it may delay the progression of motor dysfunction. The motor function in four children (26.7%) had a slight improvement 24 weeks after pantethine treatment, which was consistently with the change of score in UPDRS I-III or FM. We found that patients with lower baseline UPDRS I-III or FM scores were more likely to be assessed as “slightly improved”, which may indicate that patients with relatively mild motor handicap at baseline were more likely to improve after the treatment, and it may also be due to fluctuations in their own condition over time. Therefore, further study with longer time and larger sample size is needed.

Appropriate dose of pantethine for PKAN is unknown yet. The recommended dose for adults with hyperlipidemia is 600 mg per day (10 mg/kg per day), which is insufficient for patients with PKAN considering the concentration for working on central nervous system. Previous experience on patients with cystinosis showed the dosage at 70 mg/kg per day was well tolerated [8]. Therefore, we chose the dosage of 60 mg/kg per day in the present study. Due to limited number of the patients, we did not try different dosage. We measured the concentration of pantethine and sodium pantothenate in the plasma. We could only detect sodium pantothenate but not pantethine, which is because that pantethine was rapidly hydrolyzed to pantothenic acid and cysteamine [8]. The peak plasma concentration of the sodium pantothenate at 2 h after taking pantethine was 1.56 ± 0.91 μg/ml. The dosage of 60 mg/kg per day was well tolerated in all children during the treatment. Higher dosage is worth to be explored in future studies.

To our acknowledge, there is no promising disease-modifying therapies for PKAN yet. Therapies used in patients included deep brain stimulation (DBS), deferiprone fosmetpantotenate (RE-024), 4′-phosphopantetheine and CoA-Z. A meta-analysis on DBS [9] has shown that it could improve the dystonia movement scores in patients with PKAN one year postoperatively. Dystonia of three out of five patients improved after DBS up to 36 months postoperatively in a case series study [10]. Overall, DBS could initially improve the dystonia movement scores in some patients with PKAN but could not stop the disease progression. Thomas Klopstock et al. [11] conducted a randomized, double-blind, controlled trial to explore the safety and efficacy of deferiprone for PKAN. Seventy-six patients were recruited in the 18-month study. No obvious difference was found in the Barry-Albright Dystonia Scale (BAD) and Patients Global Impression of Improvement scores (PGI-I) between the deferiprone and placebo groups. The progression in patients switching from placebo to deferiprone reduced in the extension study, similar with the effect of pantethine in our trial. Christou et al. [12] conducted a study on a single patient with oral administration of RE-024 (Fosmetpantotenate) for 12 months. The clinical parameters improved in this patient, including UPDRS scale, Barry–Albright Dystonia Scale, and 25-ft walk test. However, there was no significant difference in primary endpoint between RE-024 groups and placebo groups in phase III clinical trials according to Retrophin’s announcement. The clinical trials about CoA-Z is ongoing. PZ-2891 treatment elevated brain CoA and improved motor function in a mouse model [13] .4′-phosphopantetheine normalized levels of the CoA, iron, and activities of mitochondrial enzymes in a mice model [14].

Current hypothesis for neurodegeneration of PKAN is that the mutations of *PANK2* result in reduction of CoA and the iron accumulation in specific brain regions. And previous studies on a *PANK2* knockout *Drosophila* model [6] showed that pantethine could increase CoA levels in tissues, which indicating that CoA may be an indicator to evaluate efficacy of pantethine in PKAN. No significant change in serum CoA level was found in the present study after 24 weeks of treatment, which was consistent with Jackowski et al, [15] indicating that serum CoA is not a suitable biomarker. The PANK2 enzyme is widely expressed in bone marrow, brain and other 25 tissues. It is localized to mitochondria of neurons in human and mouse brain, [16] CoA in mitochondria may need to be detected.

Some limitations were in the present study: (1) This is a single-arm, open-label study. The number of patients was limited. (2) The treatment duration was not long. (3) We did not try different dosage of pantethine in the study. (4) Blood concentration of sodium pantothenate was collected only at 2 h and 5 h after taking pantethine. Trough concentration just before taking medicine was not collected. (5) The UPDRS I-III and FM scores at W-24 were retrospectively scaled.

## Conclusions

In this study on 15 children with PKAN, we did not find 24 weeks of pantethine could significantly improve motor function, but we found that it may delay the progression of motor dysfunction. Patients tolerated very well to the dosage of 60 mg/kg per day. Higher dosage in larger patient samples should be studied to determine the therapeutic effects in PKAN.

## Data Availability

The datasets used and/or analyzed during the current study are available from the corresponding author on reasonable request.

## Abbreviations

PKAN: Pantothenate kinase-associated neurodegeneration
DBS: Deep brain stimulation
CoA: Coenzyme A
UPDRS: Unified Parkinson’s Disease Rating Scale
FM: Fahn–Marsden Scale
ADL: Activities of daily living
PedsQL: Pediatric Quality of Life Inventory
BAD: Barry–Albright Dystonia Scale
PGI-I: Patients Global Impression of Improvement scores
